# The Influence of Pharmacological Agents Used During General Anesthesia on the Intensity of Postoperative Pain and the Occurrence of Post-Anesthetic Delirium—A Scoping Review

**DOI:** 10.3390/jcm15051867

**Published:** 2026-02-28

**Authors:** Amelia Dąbrowska, Izabella Jadwiga Brykczyńska, Sandra Lange, Mateusz Szczupak, Sabina Krupa-Nurcek, Wioletta Mędrzycka-Dąbrowska

**Affiliations:** 1 Faculty of Medicine, Lazarski University in Warsaw, 02-662 Warsaw, Poland; 49175@lazarski.edu.pl (A.D.); 50607@lazarski.edu.pl (I.J.B.); 2 Department of Internal and Pediatric Nursing, Medical University of Gdansk, Dębinki 7, 80-211 Gdansk, Poland; langa94@gumed.edu.pl; 3 Department of Anesthesiology and Intensive Care, Copernicus Hospital, 80-803 Gdansk, Poland; szczupak.mateusz@icloud.com; 4 Department of Surgery, Faculty of Medicine, Collegium Medicum, University of Rzeszow, 35-959 Rzeszow, Poland; sabinakrupa@o2.pl; 5 Department of Anaesthesiology Nursing & Intensive Care, Faculty of Health Sciences, Medical University of Gdansk, 80-211 Gdansk, Poland

**Keywords:** anesthetics, pain, delirium, postoperative

## Abstract

**Introduction**: Postoperative delirium, including emergence agitation, is recognized in the post-anesthesia care unit as a fluctuating disturbance of attention and cognition. The current evidence examined suggests that both anesthetic agents and postoperative pain intensity may influence the risk of delirium. The aim of this review is to discuss the significance of pharmacological agents used during anesthesia and the relationship between the intensity of postoperative pain and the occurrence of postoperative delirium in patients undergoing surgical procedures, regardless of age. **Methods**: A scoping review was conducted from December 2024 to December 2025. The articles identified in each search were limited to those published between 2015 and 2025. **Results**: Agents such as dexmedetomidine, remimazolam, and magnesium sulfate were examined in the included trials and were reported to be associated with reducing the incidence and severity of postoperative delirium, particularly in pediatric and elderly patients. Analysis of clinical trial outcomes conducted in pediatric populations undergoing various surgical procedures suggests that dexmedetomidine (administered intranasally and intravenously) and alfentanil were associated with lower incidence and severity of emergence delirium compared to standard care or other agents (e.g., midazolam). Higher doses of dexmedetomidine (2 µg/kg) were reported to be associated with improved postoperative analgesia and reduced agitation, without prolonging recovery time or causing serious adverse effects. Propofol, due to its rapid metabolism, was suggested to contribute to shorter emergence times; however, its impact on cognitive function requires further investigation. Additionally, there remains a lack of agreed-upon and/or validated tools and strategies for pain assessment in patients experiencing delirium. **Conclusions**: The current evidence examined suggests that the use of intranasal dexmedetomidine at appropriate doses may be associated with reduced postoperative pain and agitation without prolonging recovery time or increasing the risk of serious adverse events. Hydromorphone was reported in the included trials to be associated with better postoperative pain control than sufentanil, whereas remimazolam, although associated with reduced delirium incidence in some trials, did not influence the length of stay in the post-anesthesia care unit. Magnesium sulfate, although not significantly affecting the incidence of delirium, was associated with alleviation of postoperative symptoms such as pain and insomnia in adult patients. Ketamine, while commonly used for analgesic therapy, did not demonstrate a consistent association with delirium prevention and, in some studies, was associated with increased neuropsychiatric events. Further research is required to more precisely define optimal perioperative delirium prevention protocols.

## 1. Introduction

Post-operative delirium (POD), sometimes also referred to as emergence agitation (EA) or anesthetic emergence agitation, can occur from anywhere between 10 and 15 min after anesthesia up until discharge from the hospital. It is commonly recognized in the post-anesthesia care unit (PACU) as a sudden, fluctuating, and usually reversible disturbance of mental status with a degree of inattention [1,2]. Agitation includes restlessness, confusion, purposeless movements, insomnia, struggling, and incoherence during the early recovery period from general anesthesia [3].

The incidence of EA varies from approximately 0.25% to 90.5% depending on age, assessment tool used, definitions, anesthetic techniques, type of surgery, and time of EA assessment during recovery. The proposed risk factors of EA include age, male sex, type of surgery, emergency operation, use of inhalational anesthetics with low blood–gas partition coefficients, long duration of surgery, anticholinergics, premedication with benzodiazepines, voiding urgency, postoperative pain, and the presence of invasive devices [3,4]. Postoperative delirium is associated with numerous adverse outcomes, including prolonged hospitalization, impaired rehabilitation, increased risk of complications, deterioration in functional status, and higher mortality rates [5,6,7]. In school-age children, postoperative cognitive dysfunction (POCD) may develop and, in the event that no commensurate remedy was made, may persist in 80% of cases for at least one month following the operation [1]. Furthermore, it constitutes a significant burden not only for patients and their families but also for healthcare professionals and the healthcare system as a whole [8]. The etiology of postoperative delirium is multifactorial and includes preoperative, intraoperative, and postoperative factors. In adults, preoperative risk factors include advanced age, neurological disorders (e.g., dementia), alcohol and psychoactive substance abuse, malnutrition, diabetes mellitus, anemia, and a history of previous delirium episodes [3,9,10]. Intraoperative factors comprise the type of surgery, the type of anesthesia, the duration of the procedure, and the extent of blood loss [11,12]. In the postoperative period, laboratory abnormalities, the number of transfused blood units, and the use of opioid analgesics play a particularly important role [9,13]. Postoperative pain is one of the major risk factors for the development of delirium. Severe pain activates the stress response and induces a neuroinflammatory cascade, which may lead to disturbances in consciousness and cognitive function [14]. In children, it is important to differentiate emergence delirium from acute postoperative pain, which is usually manifested in the child’s facial expression [5]. Effective perioperative pain management is therefore crucial for delirium prevention; however, the selection of the optimal analgesic strategy remains the subject of ongoing research. Although opioids are highly effective analgesics, they may increase the risk of delirium due to their sedative effects and influence on the cholinergic system. In contrast, regional analgesic techniques, such as nerve blocks, may reduce this risk by decreasing opioid requirements [15,16].

### Aim

The aim of this review is to discuss the significance of pharmacological agents used during anesthesia and the relationship between the intensity of postoperative pain and the occurrence of postoperative delirium in patients undergoing surgical procedures, regardless of age.

## 2. Methods

### 2.1. Study Design

The scoping review was conducted from December 2024 to December 2025. The number of articles found during each search test was limited to studies conducted between 2015 and 2025. A scoping review methodology was selected to map key concepts related to pain management and postoperative delirium [17]. Scoping reviews draw on evidence derived from a wide range of research methodologies and may also incorporate evidence from non-research sources [17,18]. The review was conducted in accordance with the methodology outlined in the Joanna Briggs Institute Manual for Scoping Reviews [17] and followed the recommendations of the Preferred Reporting Items for Systematic Reviews and Meta-Analyses extension for Scoping Reviews (PRISMA-ScR) guidelines [19] (Supplementary Materials Table S1). The Arksey and O’Malley framework was applied, which comprises five stages: (I) identifying the research questions, (II) identifying relevant studies, (III) study selection, (IV) data charting, and (V) collating, summarizing, and reporting the results [20].

### 2.2. Identifying the Research Question

To identify key aspects related to pain management strategies and the occurrence of postoperative delirium, the following research questions were formulated:

1. Can pharmacological agents used during general anesthesia prevent or exacerbate postoperative delirium?
2. What is the effectiveness of pharmacological agents used during anesthesia in preventing postoperative pain?
3. Which rating scales are most commonly used to assess delirium and pain intensity in the postoperative period?

### 2.3. Identifying Relevant Studies

Two authors (A.D. and S.L.) systematically searched the following databases: PubMed, Web of Science, and Scopus. Search strategy: adult OR pediatric AND “general anesthesia” OR anesthesia OR surgery AND “postoperative pain” OR “pain management” AND “postoperative delirium”. All publications were analyzed by title and abstract to exclude irrelevant entries. The final search was carried out in December 2025.

Any disagreements were addressed through discussions with the researchers, and by the end of the selection process, full consensus was reached regarding the articles to be included. The articles identified in each search were limited to those published between 2015 and 2025. To identify appropriate studies, we applied the population–concept–context (PCC) framework, as recommended by the Joanna Briggs Institute (JBI). Strict inclusion and exclusion criteria were followed (Table 1). Reviews were deemed eligible if all specified criteria were met.

### 2.4. Charting the Data

Data such as author, country, type of study, purpose, study group, type of anesthesia, delirium score, pain intensity score, and conclusions were extracted from all relevant studies (Table 2 and Table 3).

### 2.5. Collating, Summarizing, and Reporting the Results

After charting the data from all included articles to show the most relevant aspects of the review, a numerical analysis was performed, and the results are summarized in Table 2 and Table 3, ordered by parameter and outcome [30]. We extracted data from the included articles that matched our review question. A narrative synthesis was then applied to analyze the results of the included studies [32].

## 3. Results

### 3.1. Study Selection

**Figure 1:**
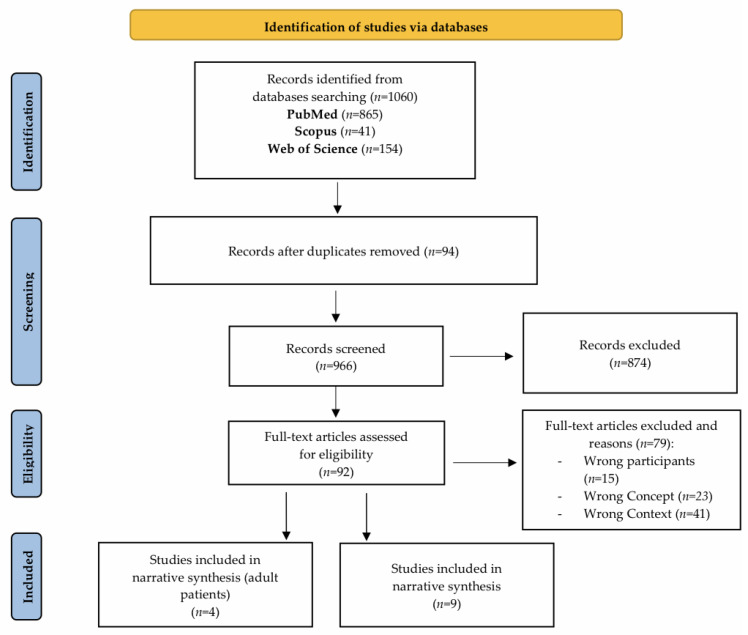
Prisma flow chart.

A total of 1060 records were initially retrieved from the three databases (PubMed, Scopus and Web of Science). After removing duplicates (94) and screening titles and abstracts (874), 92 full-text studies were assessed for eligibility. The primary reasons for exclusion were mainly due to three types of mismatch: population, concept, and context. In 15 studies, the analyzed group of participants did not meet the established criteria. In 23 studies, the concept of the studies did not directly relate to the phenomenon being analyzed, and 41 articles were excluded due to context, as the studies were conducted in an environment other than the ICU. Ultimately, the final analysis included a total of 13 articles [21,22,23,24,25,26,27,28,29,30,31,32,33] (Figure 1).

### 3.2. Study Characteristics

A total of 13 studies were included in the analysis. All included studies were randomized, of which five were double-blind, placebo-controlled randomized trials and two were prospective randomized controlled trials (RCTs). The largest number of studies was conducted in China (*n* = 7), while the remaining studies originated from Turkey (*n* = 1), Egypt (*n* = 2), South Korea (*n* = 1), and countries such as USA, Canada, India, South Korea (1) and India (*n* = 1).

The studies included both pediatric (*n* = 9) and adult (*n* = 4) populations. In the pediatric group, participants’ ages ranged from 6 months to 7 years, whereas in the adult group, ages ranged from 18 to 92 years. General anesthesia was used in 11 studies, while total intravenous anesthesia (TIVA) was applied in two studies.

The results presented in Table 4 demonstrate substantial methodological and clinical heterogeneity among the included studies, highlighting the complexity of assessing postoperative delirium and pain across different age groups. At the same time, they indicate that the effectiveness of various anesthetic techniques and pharmacological strategies has been extensively investigated in the context of pain minimization, reduction in delirium risk, and improvement of overall postoperative recovery.

## 4. Discussion

The aim of this review is to discuss the significance of pharmacological agents used during anesthesia and the intensity of postoperative pain on the occurrence of postoperative delirium in patients undergoing surgical procedures, regardless of age. The current evidence examined indicates a complex relationship between anesthetic pharmacotherapy, postoperative pain, and delirium occurrence.

Delirium in the PACU is a subtype of perioperative delirium. The difference between PACU and postoperative delirium lies primarily in the timeframe used: the former occurs in the PACU on the day of surgery, while the latter occurs after surgery. This definition can be confusing, as delirium in the PACU is an inextricable combination of delirium/agitation in the recovery phase and postoperative delirium. Agitation in the recovery phase refers to restlessness, disorientation, agitation, aimless movements, jerking, and incoherence during the early recovery period from general anesthesia. It is typically short-lived, self-limiting, and has minimal long-term sequelae. However, the presence of agitation in the recovery phase remains a strong predictor of postoperative delirium [34].

Despite the prevalence of delirium and its high associated morbidity and mortality, there is a dearth of research regarding its phenomenology in adults, and the situation for children is even worse. In the study by Turkel et al., the same symptoms of delirium were shown to occur in adults and children, which supports the clinical practice of making a diagnosis of delirium based on DSM criteria in patients of all ages [35]. Leentjens et al. found that delirium in children has a different course and symptom profile than delirium in adults and the elderly. Delirium in adults and the elderly differs only in the severity of cognitive symptoms [36]. Schieveld and Zwieten recommended developing a uniform screening tool across the age range, which could create a common diagnostic language and standardize the process of diagnosing delirium. However, developmental differences in patient populations, such as older adults with dementia or comorbidities, with their varying patterns of delirium expression, make the development of a universal tool unrealistic [37,38]. In both children and adults, similar classes of medications, primarily antipsychotics (e.g., haloperidol, quetiapine, olanzapine), are used for the pharmacological treatment of delirium. However, in pediatrics, the evidence base is limited, and a greater emphasis is placed on non-pharmacological treatment, so the indications and use of medications are not entirely consistent across age groups [39,40,41].

### 4.1. Comparison of the Effectiveness of Anesthetic Agents in Preventing Postoperative Delirium

Pharmacotherapy has been investigated as a potential strategy in preventing postoperative cognitive disorders such as delirium. Agents such as dexmedetomidine and remimazolam were examined in the included studies and were reported to be associated with reductions in the incidence and severity of postoperative delirium, particularly in pediatric and elderly patients [21,32]. Dexmedetomidine at a dose of 2 µg/kg was reported to provide effective postoperative analgesia, and its sedative properties were associated with reduced occurrence of agitation and delirium without prolonging recovery time [21]. Remimazolam, although not reducing pain intensity, was associated in pediatric trials with a lower incidence of delirium without affecting hospital discharge times [29,34]. However, some drugs, such as subanesthetic doses of ketamine, did not demonstrate a significant reduction in delirium incidence in the examined trials, suggesting that their use in this context may not provide consistent benefit [31]. In the study by Avidan et al., there was no difference in the incidence of delirium between patients in the ketamine and placebo groups, but higher doses of ketamine were associated with increased reports of hallucinations and nightmares [31].

Currently, multimodal analgesia is considered the standard approach for postoperative pain management. This strategy involves the concurrent use of different classes of analgesics with distinct mechanisms of action to achieve optimal analgesia while minimizing the doses of individual agents, thereby reducing the risk of adverse effects [22]. Combining opioids, non-steroidal anti-inflammatory drugs (NSAIDs), paracetamol, and adjuvants such as gabapentinoids (gabapentin, pregabalin) allows for more effective pain control than using a single analgesic [33]. General anesthesia combined with additional pharmacological agents, as demonstrated in Wael Fathy’s study with magnesium sulfate, shows potential in reducing delirium and insomnia [30]. The careful selection of anesthetic agents, such as midazolam, may influence patient stress levels and emergence, which may reduce the risk of cognitive complications and accelerate recovery [24].

Clinical trial data in pediatric populations undergoing various surgical procedures suggest that dexmedetomidine (intranasal and intravenous) and alfentanil were associated with lower rates of emergence delirium compared with standard methods or other agents. Intranasal dexmedetomidine was reported to be associated with lower delirium scores compared with oral midazolam, and higher doses were associated with stronger effects without increasing adverse events. Alfentanil was also reported to be associated with reduced delirium incidence without negatively affecting emergence times [23].

### 4.2. Comparison of the Effectiveness of Anesthetic Agents in Pain Prevention

Studies by Liu et al. reported no significant differences between remimazolam and propofol in terms of delirium incidence or pain scores. Propofol was suggested to facilitate shorter emergence times; however, its impact on cognitive outcomes requires further investigation [30]. Dexmedetomidine, administered intranasally or intramuscularly, was associated with reduced separation anxiety and lower postoperative pain scores, and higher doses (2 µg/kg) were reported to be associated with improved analgesia and reduced agitation without prolonging emergence or causing serious adverse events. Intramuscular dexmedetomidine (1 µg/kg) was associated with lower agitation and pain scores compared with oral gabapentin [33]. Comparisons between hydromorphone and sufentanil indicated that hydromorphone was associated with lower postoperative pain scores in children undergoing congenital defect surgeries, while both opioids had similar safety profiles [22]. Subanesthetic doses of ketamine did not demonstrate consistent reductions in pain or delirium incidence [31]. Comparisons between remimazolam and propofol showed no significant differences in the incidence or duration of postoperative delirium, pain levels, or adverse effects, suggesting similar impacts on postoperative patients [21,29].

Both dexmedetomidine and tramadol were associated with reductions in postoperative pain in children; however, dexmedetomidine was additionally associated with improved extubation quality and reduced agitation [27].

An alternative to conventional pharmacotherapy includes regional analgesic techniques, such as neuraxial anesthesia and nerve blocks, which allow effective pain control while reducing the need for centrally acting drugs [37]. Studies have shown that regional anesthesia may reduce the risk of postoperative delirium, particularly in elderly patients undergoing major surgical procedures [38].

### 4.3. Assessment of Pain Intensity and Delirium

The current evidence examined demonstrates substantial heterogeneity in the assessment tools used across studies. In adult populations, frequently reported instruments included the NRS and VAS, whereas in pediatric populations, observational scales such as FLACC and CHEOPS were commonly described. The selection of these tools was reported to vary according to patient age and communication ability, particularly in children unable to verbally report pain; the FLACC scale was also described as applicable in children with cognitive impairments. Delirium assessment methods reported in adult studies included CAM-ICU, DSM-IV criteria, RASS, and MDAS, while pediatric studies most commonly utilized the PAED scale. The reviewed studies described the use of standardized assessment instruments, with a range of methods applied to measure pain and cognitive outcomes [21,22,23,24,25,26,27,28,29,30,31,32,33]. Severe pain has been reported in prior research as being associated with an increased likelihood of postoperative delirium, with one study indicating an approximate 5.49-fold association [39]. A review by Fisher et al., which examined pain assessment in patients with delirium, identified a lack of standardized and/or validated tools and strategies for assessing pain in this population [42]. Recommendations in the literature suggest that screening for postoperative delirium be initiated early in the postoperative period [43,44,45,46,47,48].

### 4.4. Pain–Delirium Relationship

The review shows that trials focused on analgesic and sedative-analgesic agents reported associations between improved pain control and lower delirium rates [23,24,27,30,31]. A systematic review by White et al. found that pain severity was associated with a higher risk of delirium [49]. These findings indicate a potential relationship between pain intensity and cognitive outcomes, although causality cannot be established. In the study by Szczupak M et al., significant associations were found between postoperative pain intensity and the severity of delirium in cardiac surgery patients, but no similar pattern was observed in neurosurgery patients. These results emphasize the importance of systematic monitoring of pain and cognitive function in high-risk postoperative populations and indicate the need for prospective studies to elucidate the complex relationship between pain, perioperative factors, and postoperative delirium [50]. According to Sampson et al., the current evidence base for pain assessment in people with delirium is insufficient. The complex relationship between dementia, pain, and delirium warrants further research in various settings [51].

## 5. Limitations of the Review

Research on postoperative delirium has observed variations in the terminology used to describe agitation after awakening, the clinical setting (operating room, postoperative ward, and intensive care unit), and the time points after terminal anesthesia. Variability also applies to diagnostic methods for assessing both pain intensity and the occurrence of emergency delirium. Delirium due to other conditions should be considered a separate category. The postoperative period lasts from the time of surgery to the patient’s discharge from the hospital. Delirium due to other conditions should not be diagnosed separately from delirium due to awakening, although any period of lucidity after delirium due to awakening should be noted. Factors predisposing or precipitating delirium due to other conditions can be broadly divided into preoperative, intraoperative, and postoperative causes. Therefore, this different terminology may have contributed to the fact that agitation after anesthesia in adult surgical patients has not been as well studied as in children [52,53].

## 6. Conclusions

The current evidence examined suggests that intranasal dexmedetomidine at appropriate doses may be associated with reduced postoperative pain and agitation without prolonging recovery time or causing serious adverse events. Hydromorphone was reported to be associated with lower postoperative pain scores compared with sufentanil. Remimazolam was associated with reduced emergence delirium in pediatric trials but did not influence PACU length of stay. Magnesium sulfate was associated with reduced postoperative pain and insomnia in adults, although no consistent association with delirium incidence was observed. Ketamine did not demonstrate consistent benefit in delirium prevention and was associated in some studies with neuropsychiatric adverse effects.

There remains a lack of standardized tools and strategies for pain assessment in patients with delirium. The current literature suggests that inadequately controlled pain may be associated with increased delirium risk. These findings support consideration of individualized anesthetic strategies and multimodal analgesia approaches. Further research is required to more precisely define optimal perioperative delirium prevention protocols.

## Figures and Tables

**Table 1:** PCC framework, inclusion and exclusion criteria, and search strategies.

	Inclusion Criteria	Exclusion Criteria
Participants (P)	Patients aged 6 months to 95 years undergoing various surgical procedures.	No data were available for patients who did not undergo surgical procedures.
Concept (C)	Pharmacological interventions administered intraoperatively	No intraoperative pharmacotherapy related to delirium or pain
Context (C)	Surgical procedures requiring general anesthesia, with assessment of the effectiveness of various agents in reducing postoperative delirium and pain	Absence of anesthesia or presence of other procedure-related diseases
Study Type (S)	double-blind placebo-controlled randomized trial, prospective randomized controlled trial, randomized controlled trial	Other studies
Years considered/Time period	All evidence published in the last 10 years, period 2015–2025	Publications published before 2015
Language	English	Other languages
Databases	MEDLINE (PubMed), Web of Science (WoS), Scopus	Other databases
Search strategy	PubMed: (((adult OR pediatric) AND (general anesthesia OR anesthesia OR surgery)) AND (postoperative pain OR pain management)) AND (postoperative delirium) Results: **865** Limits: Language and years WoS: (((TS = (adult OR pediatric)) AND TS = (“general anesthesia” OR anesthesia OR surgery)) AND TS = (“postoperative pain” OR “pain management”)) AND TS = (“postoperative delirium”) Results: **41** Limits: Language and years Scopus: (ALL (adult OR pediatric) AND TITLE-ABS-KEY (general AND anesthesia OR anesthesia OR surgery) AND TITLE-ABS-KEY (postoperative AND pain OR pain AND management) AND TITLE-ABS-KEY (postoperative AND delirium)) Results: **154** Limits: Language and years	n/a
Keywords	“delirium”, “pain”, “general anesthesia”, “postoperative pain”, “postoperative delirium”	n/a

n/a—not applicable.

**Table 2:** Characteristics of the included studies in the 1- to 10-year group.

Author	Country	Type of Study	Type of Procedure	Purpose of the Study	Study Group	Type of Anesthesia	Medicines/ Group Size	Delirium Assessment	Scales Used to Assess Pain Intensity	Conclusions
Li L.Q. et al. 2018 [21]	China	DB-RCT	Adenoidectomy with or without tonsillectomy	This study aimed to investigate the effects of different doses of intranasal dexmedetomidine on the preoperative sedation and postoperative agitation in pediatric patients with TIVA for adenoidectomy with or without tonsillectomy.	■ Aged 2 to 7 years	TIVA	Twenty-five to 40 min before surgery, the D1 (*n* = 30) and D2 (*n* = 30) groups received intranasally dexmedetomidine 1 μg kg^−1^ or 2 μg kg^−1^, respectively, while the S (*n* = 30) group received saline of the same volume.	PAED As for the PAED scale scores, there was a significant difference between the D2 and S groups (*p* = 0.029), while there was no significant difference between the D1 and S groups (*p* = 0.087), or between the D1 and D2 groups (*p* = 0.890)	CHEOPS There was a significant difference in CHEOPS scores between the D2 and S groups (*p* = 0.013), while there was no significant difference between the D1 and S groups (*p* = 0.483), or between the D1 and D2 groups (*p* = 0.199)	The intranasal dexmedetomidine of 1 or 2 μg kg^−1^ 25 to 40 min before induction of anesthesia could deliver effective preoperative sedation, reducing the children’s distress of separation from parents. Moreover, intranasal dexmedetomidine of 2 μg kg^−1^ could deliver more effective postoperative analgesia and reduce postoperative agitation, without prolonging postoperative recovery or causing severe adverse events.
Pan Y. et al. 2021 [22]	China	RCT	Surgical repair of structural congenital malformations in children	To compare the effectiveness of hydromorphone hydrochloride and sufentanil, combined with flurbiprofen axetil, for postoperative analgesia in pediatric patients.	■ Aged 6 months to 3 years	TIVA	Patients were randomized 1:1:1 into 3 groups: hydromorphone hydrochloride 0.1 mg/kg (H1, *n* = 74), hydromorphone hydrochloride 0.2 mg/kg (H2, *n* = 74), or sufentanil 1.5 µg/kg (S, *n* = 74).	PAED There were no significant differences in the PAED scale.	FLACC The FLACC pain score was significantly lower in patients who received hydromorphone hydrochloride 0.1 mg/kg (*p* < 0.01) or hydromorphone hydrochloride 0.2 mg/kg (*p* = 0.01) compared to patients who received sufentanil 1.5 μg/kg. There was no significant difference in FLACC pain score in patients who received hydromorphone hydrochloride 0.1 mg/kg or hydromorphone hydrochloride 0.2 mg/kg	Hydromorphone hydrochloride is a more effective analgesic than sufentanil for postoperative pain in pediatric patients following surgical repair of a structural congenital malformation under general anesthesia Hydromorphone hydrochloride and sufentanil had similar safety profiles in this patient population (side effects of the pain control methods were not evaluated).
Zhao N. et al. 2022 [23]	China	RCT	ambulatory dental treatment	The study aimed to investigate the effects of alfentanil intravenous infusion on emergence delirium and other perioperative complications.	■ Aged 3–6 years.	General anesthesia	Alfentanil was administered as a continuous infusion from the beginning to the end of the operation at two doses: group Alf2 (*n* = 57) received 0.2 μg/kg/ min of alfentanil and group Alf4 (*n* = 57) received 0.4 μg/kg/min of alfentanil. The control group was given saline (group Sal) (*n* = 57).	PAED There were no significant differences between group Alf2 (6.4 ± 3.5) and group Alf4 (5.8 ± 3.8) PAED scores (mean difference 0.579 [95% confidence interval {CI} −1.279 to 2.437], *p* = 0.742), and scores were significantly lower than those for the saline control patients (9.6 ± 5.1)	mCHEOPS Pain scores in group Alf2 (2.4 ± 1.5) and group Alf4 (2.1 ± 1.6) were similar (mean difference 0.386 [95% CI −0.425 to 1.197], *p* = 0.5) and were significantly lower than those in group Sal (4.1 ± 2.3) (mean difference −1.665 [95% CI −2.472 to −0.858], *p* < 0.01, and mean difference −2.051 [95% CI −2.858 to −1.244], *p* < 0.01, respectively)	Intravenous infusion of 0.2 μg/kg/min and 0.4 μg/kg/min alfentanil decreased the incidence of emergence delirium in the post-anesthesia care unit.
Yao Y. et al. 2020 [24]	China	RCT	strabismus surgery	To identify the effectiveness of pre-operative intranasal dexmedetomidine for emergence delirium in the pediatric patient population following general anesthesia	■ Aged 3–6 years	General anesthesia	Patients in Group D (*n* = 52) received intranasal dexmedetomidine (2 mg kg^−1^) 45 min and oral 0.9% saline 30 min before induction of anesthesia. Patients in Group M (*n* = 50) received intranasal 0.9% saline 45 min and oral midazolam (0.5 mg kg^−1^) 30 min before induction of anesthesia. Patients in Group P (*n* = 51) received intranasal 0.9% saline 45 min and oral 0.9% saline 30 min before the installation of anesthesia.	PAED The incidence of emergence delirium in Group D was significantly lower than that in Group M (*p* < 0.001, RR ¼ 0.262, 95% CI 0.116 to 0.592) and in Group P (*p* < 0.001, RR ¼ 0.235, 95% CI 0.105 to 0.525). There were no significant differences between Groups M and P (P ¼ 0.256, RR ¼ 0.898, 95% CI 0.59 to 1.366).	CHEOPS There were no significant between-group differences in the postoperative pain scores (modi- fied CHEOPS) in the PACU (P ¼ 0.269).	Intranasal dexmedetomidine was more effective than oral midazolam in reducing the incidence of wakefulness delirium and improving the quality of awakening in children without prolonging the awakening time or increasing the adverse events
Koceroglu I. et al. 2020 [25]	Turkey	RCT	adenotonsillotomy	Comparison of the effects of dexmedetomidine and tramadol on postoperative pain, agitation and quality of extubation in children after adenotonsillectomy	■ Aged 2–9 years	General anesthesia	Patients randomized into the dexmedetomidine group (group D, *n* = 30) and the tramadol group (group T, *n* = 30) received 1 μg/kg dexmedetomidine or 1.5 mg/kg tramadol, respectively.	PPSS	SAS	Both dexmedetomidine and tramadol effectively reduced postoperative pain and agitation, with dexmedetomidine providing better extubation quality.
								Patients in the dexmedetomidine group had significantly lower post-operative PPSS and Riker SAS scores than patients in the tramadol group.		
Abdel-Rahman K.A. et al. 2018 [26]	Egypt	RCT	strabismus surgery	Assessment of the effect of two different doses of dexmedetomidine on the incidence of agitation and delirium following strabismus surgery in children	■ Aged 3–6 years underwent strabismus surgery	General anesthesia	The first group was high Dex group (*n* = 30), in which children received 0.5 μg kg^−1^ of dexmedetomidine diluted in 10 mL of normal saline. The second group is low Dex group (*n* = 30) in which children received 0.25 μg kg^−1^ of dexmedetomidine diluted in 10 mL of normal saline.	PAED Regarding the number of patients who suffered from emergence agitation with PAED score > 10, there was 1 (3.3%) case in high Dex group, 4 (13.3%) cases in low Dex group, and 10 (33.3%) cases in the placebo group. A significant difference was found in the incidence of EA between the three studied groups	FLACC Maximum FLACC score measured in the median PACU was significantly lower in high Dex groups (1.5), while in the low Dex group it was (2) compared to placebo group (5), with no significant difference between the two Dex groups	Both doses of dexmedetomidine reduced the incidence of agitation and delirium, with the 0.5 μg/kg dose being more effective.
Sun Y. et al. 2017 [27]	China	RCT	laparoscopic inguinal hernia repair	Evaluation of the efficacy of dexmedetomidine in preventing agitation and delirium in children after laparoscopic hernioplasty	■ Aged 3–7 years	General anesthesia	Children were randomized 1:1 to one of four groups (*n* ¼ 25/group) using a computer- generated random numbers table: a control group (group C, which received saline) and three treatment groups, which were given different Dex doses (D1, D2, and D3 groups receiving doses of 0.25, 0.5, and 1.0 mg/kg, respectively)	Five-point scale for emergence agitation and delirium The frequency of EA shows a declining trend with increasing doses of Dex (P ¼ 0.001)	CHIPPS Dex reduced the pain of the children, as shown by the CHIPPS score (*p* < 0.001), with Dex at 0.5 and 1.0 mg/kg having better efficacy than at 0.25 mg/kg.	Dexmedetomidine significantly reduced the frequency and severity of post-arousal agitation and delirium. It also reduced pain and improved recovery.
Lee J.L. et al. 2020 [28]	South Korea	RCT	strabismus surgery	Evaluation of the effects of magnesium supplementation during general anesthesia on emergence delirium and postoperative pain in children undergoing strabismus surgery	■ 2 to 5 years	General anesthesia with Sevoflurane	The magnesium group received an initial loading dose of 30 mg/kg magnesium sulfate over 10 min and, then, continuous infusion of 10 mg/kg per h until 10 min before the end of the surgery. The control group received an equal volume of normal saline via the same regimen.	PAED There were 26 of 33 (78.8%) and 27 of 32 (84.4%) children with emergence delirium in the control and the magnesium groups, respectively (odds ratio 0.69, 95% CI 0.19–2.44; *p* = 0.561).	CHEOPS The PAED and pain scores of the two groups did not differ significantly.	Magnesium supplementation had no significant effect on emergence agitation or postoperative pain in children who had undergone strabismus surgery.
Yang X. et al. 2022 [29]	China	DB-RCT	Tonsillectomy and adenoidectomy	To identify the effectiveness of remimazolam at the end of tonsillectomy and adenoidectomy for preventing emergence delirium in children under Sevoflurane anesthesia	■ Aged 3–7 years	General anesthesia with Sevoflurane	Patients were randomly assigned to receive either remimazolam 0.2 mg kg ^−1^ (intervention, *n* = 52) or 0.9% normal saline (control, *n* = 52) at the end of the procedure.	PAED The peak PAED scores (median [IQR]) were lower in the remimazolam group than in the saline group (7 [6–8] vs. 9 [8–11], *p* < 0.001), with a median difference of −2 (95% CI −1 to −3). Emergence delirium occurred in 6 of 51 (12%) patients receiving remimazolam versus 22 of 50 (44%) patients receiving saline (risk difference 32% [95% CI, 16% to 49%], relative risk = 0.27 [95% CI, 0.12 to 0.60]; *p* < 0.001)	FLACC The peak FLACC pain score was 2.0 (IQR, 1.0–2.0) in the remimazolam group and 2.0 (IQR, 2.0–2.0) in the control group.	Administration of remimazolam at a dose of 0.2 mg/kg at the end of surgery reduces the incidence of recovery delirium without delaying discharge from the postoperative care unit (PACU) in children after tonsillectomy and adenoidectomy performed under Sevoflurane anesthesia.

Legend: Double-blind randomized controlled trial (DB-RCT); Randomized controlled trial (RCT); Clinical Trial (CT); Total Intravenous Anesthesia (TIVA); Pediatric Anesthesia Emergence Delirium (PAED); modified Children’s Hospital of Eastern Ontario Pain Scale (mCHEOPS); Children’s and Infants’ Postoperative Pain Scale (CHIPPS); Children’s Hospital of Eastern Ontario Pain Scale (CHEOPS); Legs, Activity, Cry, Consolability (FLACC); Dexmedetomidine (Dex); Post-operative pain was assessed with the pain point system scale (PPSS); Riker Sedation–Agitation Scale (SAS).

**Table 3:** Characteristics of the included studies in the 18- to 85-year-old group.

Author	Country	Type of Study	Type of Prodecure	Purpose of the Study	Study Group	Type of Anesthesia	Medicines	Delirium Assessment	Scales Used to Assess Pain Intensity	Conclusions
Liu T. et al. 2024 [30]	China	RCT	Colon cancer	Comparison of the effects of remimazolam and propofol on the occurrence and duration of postoperative delirium in elderly patients undergoing radical resection of colon cancer	■ aged ≥ 65 years	General anesthesia	remimazolam group (group R *n* = 50) propofol group (group P *n* = 50)	CAM-ICU There was no statistically significant difference in the incidence and duration of postoperative delirium between the 2 groups (*p* > 0.05).	VAS There were no significant differences in VAS scores, remifentanil consumption, and adverse reactions, including nausea and vomiting, hypoxemia, and respiratory depression between the 2 groups (*p* > 0.05)	Remimazolam did not reduce or increase the frequency or duration of postoperative delirium compared with propofol. There were no significant differences in pain or the incidence of adverse events.
Avidan MS. et al. 2017 [31]	USA Canada India South Korea	DB-RCT	Cardiac and noncardiac surgery	Evaluation of the effect of intraoperative ketamine on the incidence of postoperative delirium and its impact on pain and opioid injury	■ Age of 60–95 years	General anesthesia and regional	(1:1:1 ratio −0.5 mg/kg ketamine [Lo-K *n* = 227]: 1 mg/kg ketamine [Hi-K *n* = 223]: saline placebo [P *n* = 222])	CAM CAM-ICU DSM-IV The incidence of delirium over postoperative days 1 to 3 was 19.82% in group P, 17.65% in group Lo-K, and 21.30% in group Hi-K. For the primary outcome of the PODCAST study, i.e., postoperative delirium incidence in the combined ketamine groups compared with those who received placebo, there was no difference found (19.45%).	BPS, BPS-IN VAS By VAS measurements, there were no apparent differences among the three groups in pain at any of the postoperative time points.	The administration of a subanaesthetic ketamine dose during surgery is not useful in preventing postoperative delirium (primary outcome) or reducing postoperative pain
Fathy W. et al. 2024 [32]	India	RCT	Lumbar fixation	To evaluate the effect of intra- operative administration of Magnesium sulfate on the occurrence of post-operative delirium and insomnia	■ Age from 42 to 46 years	General anesthesia	Group Magnesium sulfate (*n* = 40) 30 mg/kg was administered over 10 min followed by as a maintenance dose of 10 mg/kg/h Control group (*n* = 40) general anesthesia only.	MDAS Post-operative MDAS scores were similar between Magnesium sulfate groups and control group {mean difference −0.85 (95% CI −2.2 to 0.5)}	VAS Magnesium sulfate group had lower post-operative VAS score {mean difference − 1.3 (95% CI −2.3 to −0.3)}.	Magnesium sulfate administration reduced the severity of postoperative insomnia and pain. No significant effect was observed on the occurrence of postoperative delirium.
Abdelaziz TSA. et al. 2025 [33]	Egypt	DB-RCT	Rhinoplasty	To evaluate and compare the effect of preoperative premedication with intramuscular dexmedetomidine and oral gabapentin on the incidence and severity of post-anesthetic agitation (EA) and postoperative pain in patients undergoing rhinoplasty.	■ Age 18–40 years old	General anesthesia	Group C, *n* = 51 (the control group), did not receive premedication. Group D *n* = 51 (dexmedetomidine group) received (1 µg/kg) intramuscular dexmedetomidine 30 min before the operation. Group G *n* = 51 (gabapentin group) received 600 mg of gabapentin 30 min before the operation by mouth.	RSAS The results showed statistically significant differences in EA scores (*p* value 0.002) and midazolam consumption (*p* value 0.01) with the lowest values in Group D. Moreover, the incidence of EA was 17.6% (9/51) in Group D, 41.2% (21/51) in Group G, and 56.9% (29/51) in Group C with a *p*-value < 0.001	VAS There were significant differences in VAS scores at 4, 8, and 12 h with the highest median (range) values in group C 4(3–6) in comparison to group D 2(1–3) and group G 2(1–3) and (*p*-value < 0.001)	Intramuscular premedication with dexmedetomidine (1 µg/kg) was more effective than oral gabapentin (600 mg) in reducing the frequency and severity of EA and in reducing postoperative pain.

Legenda: Double-blind randomized controlled trial (DB-RCT); Randomized controlled trial (RCT); Clinical Trial (CT); Intervention (I); Behavioral Pain Scale (BPS); Behavioral Pain Scale for the Non-Intubated patient (BPS-NI); Richmond Agitation–Sedation Scale (RASS); Confusion Assessment Method for the Intensive Care Unit (CAM-ICU); Numeric Rating Scale (NRS); Visual Analogy Scale (VAS); Diagnostic and Statistical Manual of Mental Disorders, Fourth Edition (DSM-IV); Ryerson Social Anxiety Scales (RSAS); Confusion Assessment Method (CAM); Memorial Delirium Assessment Scale (MDAS).

**Table 4:** Summary of studies included in the review.

Parameter		Results
Types of study		Double-blind placebo-controlled randomized trial: 4 Randomized controlled trials: 9
Country		China: 7
		Turkey: 1
		Egypt: 2
		South Korea: 1
		India: 1
		USA, Canada, India, South Korea: 1
Age		Kids: from 6 months to 10 years Adults: from 18 years to 92 years of age
Type of anesthesia		TIVA: 2 General anesthesia:11
Methods of assessing delirium	kids	PAED, PPSS, 5-point scale for emergency agitation and delirium
	adults	CAM, CAM-ICU, DSM-IV, MDAS, RSAS
Pain assessment methods	kids	CHEOPS, FLACC, mCHEOPS, SAS
	adults	VAS, BPS, BPS-IN
Pharmacological intervention	kids	Dexmedetomidine, Hydromorphone hydrochloride, Midazolam, Tramadol, Alfentanil, Magnesium sulfate, Remimazolam
	adults	Remimazolam, Propofol, Ketamine, Magnesium sulfate, Dexmedetomidine, Gabapentin

Confusion Assessment Method (CAM); **Confusion Assessment Method for the Intensive Care Unit (CAM-ICU);** Diagnostic and Statistical Manual of Mental Disorders, Fourth Edition (DSM-IV); Memorial Delirium Assessment Scale (MDAS); Ryerson Social Anxiety Scales (RSAS); Children’s Hospital of Eastern Ontario Pain Scale (CHEOPS); Legs, Activity, Cry, Consolability (FLACC); modified Children’s Hospital of Eastern Ontario Pain Scale (mCHEOPS); Riker Sedation–Agitation Scale (SAS); Visual Analogy Scale (VAS); Behavioral Pain Scale (BPS); Behavioral Pain Scale for the Non-Intubated patient (BPS-NI).

## Data Availability

The authors declare that the data of this research are available from the corresponding author on request.

## Supplementary Materials

The following supporting information can be downloaded at https://www.mdpi.com/article/10.3390/jcm15051867/s1, Table S1: Scoping Reviews (PRISMA-ScR) Checklist.
